# Friends’ Experiences of Bereavement Following a Drug-Related Death: A Qualitative Study

**DOI:** 10.3390/bs16081399

**Published:** 2026-08-15

**Authors:** Sari Kaarina Lindeman, Lillian Bruland Selseng, Kristine Berg Titlestad

**Affiliations:** 1 Department of Welfare and Participation, Faculty of Health and Social Sciences, Western Norway University of Applied Sciences, Campus Bergen, 5063 Bergen, Norway; kristine.berg.titlestad@hvl.no; 2 Department of Welfare and Participation, Faculty of Health and Social Sciences, Western Norway University of Applied Sciences, Campus Sogndal, 6856 Sogndal, Norway; lillian.bruland.selseng@hvl.no

**Keywords:** friend, friendship, bereavement, drug-related death, disenfranchised grief, reflexive thematic analysis

## Abstract

**Introduction:** Friendship bereavement is under-recognised, and grief following a drug-related death may be compounded by stigma and limited social validation. This study examines how bereaved friends understand and experience grief, focusing on how relational dynamics, social context, and existential concerns shape bereavement. **Methods**: We undertook a qualitative study embedded in a Norwegian project on drug-related death bereavement. Fourteen bereaved friends (8 men, 6 women; 21–54 years) completed semi-structured interviews. Data were audio-recorded, transcribed verbatim, de-identified, and analysed using reflexive thematic analysis within an interpretive qualitative tradition. Ethics approval and written informed consent were obtained. **Results:** From the analysis, four interconnected themes were generated: (1) Meaningful bonds under pressure: friendships were deeply significant yet increasingly strained as substance use escalated, generating ethical and emotional labour and fluctuating closeness. (2) The struggle for recognition: friends often occupied marginal positions in grief hierarchies; family inclusion was profoundly validating, whereas absent recognition fostered isolation. (3) Moral ties and relational guilt: post-loss self-scrutiny centred on whether to remain close, step back, or repeatedly recalibrate involvement. (4) An existential turning point: losing a close friend with whom one shared important life experiences prompted reflection on vulnerability, mortality, and life direction. **Discussion:** Bereavement following drug-related death is not only emotional but socially negotiated and existentially charged. Practice may benefit from recognising friends as mourners and more deliberately including them in rituals and communication where appropriate; research should treat friends as a distinct group and examine how pre-loss relational labour and stigma shape grief trajectories. **Highlights:** 1. Friendship as an under-examined context of drug-death bereavement: This study advances limited scholarship by showing how friends navigate grief after a drug death, where relational strain, stigma, and emotional and ethical responsibilities profoundly shape the bereavement experience; 2. Recognition of friends as bereaved is crucial: Friends grieving a drug death are often marginalised within social grief hierarchies. When families and services acknowledge the friendship, it affirms their loss and may reduce isolation and stigma; 3. Losing a close friend with whom one shares important life experiences carries strong existential weight: The death of a close friend prompts deep reflection on vulnerability, life direction, and personal responsibility, making grief both emotionally demanding and existentially significant.

## 1. Background

Friendships are central and emotionally meaningful relationships across the lifespan, yet grief following the death of a friend has received limited recognition within bereavement research (Chen et al., 2025). Dunbar (2018, p. 32) defines friends as “the people who we share our lives with, in a way that is more than just the casual meeting of strangers. Friends are the people whom we make an effort to maintain contact with, and to whom we feel an emotional bond”. Unlike family ties, friendships are voluntary, continually negotiated, and grounded in reciprocity and perceived relational quality rather than legal or genetic obligations (Dunbar & Machin, 2014; Roberts & Dunbar, 2011). These characteristics make friendships both meaningful and structurally vulnerable, because they depend on ongoing mutual investment, lack formalised social recognition, and may be more susceptible to strain during periods of life difficulty (Dunbar, 2018).

Research on friend bereavement is emerging but remains sparse. A recent review identified only a small number of empirical studies, even among adolescents and young adults, where peer relationships are central (Chen et al., 2025). Across these studies, friend bereavement is portrayed as intense and enduring, sometimes more disruptive than certain familial losses.

A large-scale longitudinal study further demonstrates long-lasting declines in psychological health, vitality, and social functioning (Liu et al., 2019). Despite these impacts, culturally shaped grief hierarchy continues to position friends as secondary mourners, increasing the likelihood of disenfranchised grief, grief that is unrecognised, minimised, or socially unsupported (Doka, 2002; Johnsen & Dyregrov, 2016; Peskin, 2019).

Drug-related deaths fall under ICD-10 external cause codes and are classified as unnatural deaths (World Health Organization, 2026). Sudden and unnatural losses are associated with more intense and complex grief reactions (Djelantik et al., 2020). In the context of drug-related death, these challenges may be further amplified. Research on families and friends bereaved by drug-related death highlights stigma, self-stigma, and associative stigma, where negative societal perceptions of illegal substance use extend to the bereaved themselves (O’Callaghan & Lambert, 2024; Stout & Fleury-Steiner, 2026). Studies document relational strain, shifting degrees of closeness and distance, and the emotional and ethical labour involved in supporting someone with substance-use difficulties (Løberg et al., 2022; Titlestad et al., 2021b). Qualitative analyses on bereaved friends and partners with lived experience of drug use describe stigma, limited recognition, and barriers to participating in rituals and receiving support (Selseng et al., 2023), showing how friends can be positioned at the margins of socially validated grief.

Qualitative findings demonstrate that friends often play a deeply valued role for families during bereavement. Parents describe the deceased’s friends as offering authenticity, shared memories, and forms of emotional expression that they find particularly meaningful (Titlestad et al., 2020). Yet, despite this emotional significance, friends may still struggle to claim a legitimate space for their grief, particularly when social norms prioritise family members as the primary bereaved (Chen et al., 2025).

Taken together, existing research shows both the importance of friend bonds and a notable gap in understanding how nonfamilial relationships are affected by drug-related death. The present study addresses this gap by exploring how drug-death bereavement is experienced in the context of friendship.

## 2. Method

A values-based reporting approach was applied in writing the methods section, guided by the Reporting Guidelines for Qualitative Research (see Braun & Clarke, 2025).

### 2.1. Methodological Orientation, Researcher Positioning, and Reflexivity

An interpretive qualitative orientation was applied, in which knowledge is understood as situated and co-constructed through narrative expression and researcher interpretation (Malterud, 2001). Themes were therefore treated as researcher-generated interpretations of shared meaning, rather than entities assumed to pre-exist within the data. Our backgrounds in bereavement research, the substance-use field, and the health and social sciences shaped how we approached the topic, interacted with participants, and interpreted the material. Researcher influence was viewed as inherent, and reflexivity was maintained throughout the study through ongoing analytic memoing and regular discussions among the three authors regarding assumptions, decisions, and interpretive moves.

### 2.2. Context and Design

The present study forms part of the larger Norwegian Drug-Death Related Bereavement and Recovery (END) project, which seeks to understand how bereaved family members and friends experience, navigate, and make sense of life following a drug-related death. The project includes both survey and interview studies, and the interviews analysed in the present article represent the friend-bereavement component of this larger research project. We conducted qualitative interviews with bereaved friends. Parts of the interview material used in this article overlap with data used in previous analyses focusing on bereaved friends and partners with lived experience of substance use (Selseng et al., 2023, 2024, 2025). Of the 14 participants included in the present study, 10 had contributed interview data to one or more of these previous analyses, while four participants had not been included in the earlier publications because they had no history of substance use. Three participants included in previous studies were not included in the present analysis because the current study focused specifically on friendship bereavement rather than ongoing intimate partner relationships. The earlier publications examined bereavement in the context of participants’ own substance use, with particular attention to grief processes, support experiences, and everyday life after loss. In contrast, the present article is the first study based on the complete sample of bereaved friends within the END project, including participants both with and without experiences of substance use. Furthermore, while previous analyses focused on the intersection between bereavement and participants’ own substance use, the present article takes friendship itself as the primary analytical focus. The analysis therefore explores how friendship shapes experiences of loss, recognition, relational responsibility, guilt, and existential meaning-making following a drug-related death.

### 2.3. Recruitment and Participants

We purposively recruited bereaved friends from across Norway to enable rich, relevant, and diverse accounts of the phenomenon. The aim was to recruit friends both without substance-use challenges and with lived experience of such challenges, whether current or in the past. Participants were included based on self-identification as bereaved friends. Individuals who were in ongoing intimate partner relationships with the deceased were not included. In a few cases, participants had previously been intimate partners, but at the time of death both understood and described their relationship as a friendship. Participants were recruited through several routes. Some participants were identified through the END project survey and had consented to be contacted for follow-up interviews. Additional participants were recruited through recommendations from other bereaved participants, including family members who informed friends about the study, as well as through snowball sampling and professional networks. Some individuals who had consented to follow-up contact through the END survey could not be reached when interview recruitment took place. Because recruitment occurred through several overlapping referral pathways, detailed records of the total number of individuals approached and the number who declined participation were not available. In total, 14 bereaved persons took part in the study (8 men, 6 women) (Table 1). Participants were 21–54 years, and the deceased ranged in age from approximately 20 to 40 years at the time of death. Time since the loss varied widely, from one year to 36 years, reflecting both recent and more distant bereavement experiences. Ten participants reported current or previous substance-use experience, while four reported no history of substance use.

### 2.4. Data Generation and Data Handling

Semi-structured, individual interviews were conducted from July 2019 to October 2020 at locations chosen by participants. All but one interview was conducted by the first and second authors. Interviews lasted ≈90 min, were audio-recorded, and transcribed verbatim by an experienced secretary.

The interview guide was co-constructed in the END project group and covered: (1) the time before the death, (2) the loss, (3) stigma and self-stigma, (4) help, support, and coping, and (5) post-traumatic growth. The flexible format allowed participants to narrate their experiences across past, present, and future in their own terms, aligning with an interpretive orientation in which meaning-making is central.

Transcripts were de-identified prior to analysis. All participant names used in quotations and participant descriptions are pseudonyms assigned by the researchers to protect anonymity and confidentiality. Quotations used for publication were translated from Norwegian into English, with attention to preserving meaning, and minor edits were made for readability.

### 2.5. Analytic Approach: Reflexive Thematic Analysis

We conducted reflexive thematic analysis (RTA), an iterative and interpretative analytic process (see Braun & Clarke, 2022). Initial coding was undertaken by the first author in Norwegian. The second author had also read all interview transcripts. Throughout the analytic process, analytic memos were developed and preliminary interpretations were discussed repeatedly among all authors. These discussions contributed to the refinement of themes, consideration of alternative interpretations, and development of the final analytic narrative. The workflow involved: (a) familiarisation through repeated, immersive reading; (b) early writing and initial coding oriented toward meaning, context, and nuance; (c) active generation of candidate patterns of shared meaning; (d) iterative review, refinement, and naming of themes through reflexive dialogue within the author team; and (e) development of a coherent analytic account supported by illustrative data extracts. Throughout, themes were understood as interpretive stories about the data. We did not use inter-rater reliability, consensus coding, or saturation claims, which are not consistent with the epistemology of reflexive thematic analysis.

### 2.6. Ethical Considerations

The study conformed to the Declaration of Helsinki and received approval from the Norwegian Regional Committees for Medical and Health Research Ethics (REK Vest) (ref. 2017/2486). Participation was voluntary, with written informed consent and the right to withdraw without consequences. We anonymised transcripts and stored data securely on the institution’s research server. In line with guidance for research with vulnerable populations (see Dyregrov, 2004), the project leader ensured that support options were available to participants after interviews, upon request.

## 3. Results

We generated four interconnected themes with related codes: (1) Meaningful bonds under pressure; (2) The struggle for recognition; (3) Moral ties and relational guilt; (4) An existential turning point. The four themes together illuminate how friends experienced and made sense of bereavement following a drug-related death. The first concerns the meaning and complexity of the friendship. The second describes the struggle for recognition as bereaved friends. The third highlights experiences of relational guilt. The fourth shows how the loss prompted existential reflection. Together, the themes illustrate how grief was shaped by relational, social, and existential concerns.

### 3.1. Meaningful Bonds Under Pressure

This theme explores how participants understood their relationship with the deceased and how it shaped their experience of bereavement. Across interviews, friendships were described as deeply meaningful, frequently formed early in life, and closely tied to participants’ identities. These long-standing ties underpinned the intensity of the grief. Jonas, for instance, had known his best friend “since we were basically babies,” and described the loss as akin to losing a younger brother. Others described adolescent and early adult friendships marked by humour, trust, and shared experiences. As the deceased’s substance use became more prominent, many friendships moved through periods where reciprocity was strained, and concern, responsibility, and ambivalence became more central. Participants described caring deeply while feeling fear, exhaustion, and a constant need to renegotiate how to stay present without becoming overwhelmed. Such shifts were crucial for how participants later made sense of the bereavement. Participants described different relational experiences depending on the circumstances of the friendship. Some participants with current or previous substance-use experience described periods of increased everyday closeness, although different attitudes towards substance use could introduce tensions. Several participants without a history of substance use described how the growing centrality of the deceased’s substance-use problems made contact less predictable and emotionally demanding. Some participants created distance for self-protection, whereas others stayed close out of loyalty and concern that withdrawing might make things worse. Nina captured this tension:

Most others who had known her earlier said things like, why do you even bother keeping in touch with her? You should just cut her out. And I thought, if she dies, I don’t want to be left knowing that I didn’t try, and that is why I didn’t end the contact. But I can’t say that our friendship gave me anything, at least not in the last year. It felt more like an obligation.

In such accounts, friendship shifts from reciprocal companionship toward a moral and emotional labour: vigilance, worry, and the practicalities of being “on call,” interspersed with brief moments of warmth that reaffirmed why the bond mattered.

This interplay of closeness and difficulty was a recurrent pattern. Participants described the unease of answering the phone, fearing bad news, and constantly balancing boundaries to remain a “good friend” under difficult circumstances. Even when contact had become sporadic, many still hoped the relationship might stabilise. Having negotiated closeness and boundaries for so long, the death marked not only a personal loss but also the end of an unfinished relational project, leaving them with the weight of earlier decisions.

Despite the challenges, participants recalled moments of joy, humour, and genuine connection that helped them remember their friend beyond substance use. These memories allowed them to grieve the person rather than the problems, and many felt the core of the friendship remained. Their relationships combined closeness with substantial emotional effort, shaping a bereavement involving sadness, relief, and gratitude. Their bereavement involved missing the person they had known, but also the future they had once hoped the relationship might return to.

### 3.2. The Struggle for Recognition

Across interviews, participants described a frequently marginalised position as bereaved friends, where drug-related loss raised not only emotion but questions of legitimacy, belonging, and the right to grieve. Many weighed how much space their grief could take, unsure whether others saw the friendship as significant enough, reflecting implicit norms about who counts as a legitimate mourner.

Participants sensed an unspoken hierarchy placing friends below immediate family, communicated through social cues, silences, and funeral rituals. As Vegard expressed:

I somehow felt that I didn’t really have the right to be sad. We were friends, and I cared a lot about him, and we were close. But he had a younger brother, he had a mother and a father, cousins, and people he was with every day—People who, in a way, had more right to be sad than I did.

His description illustrates how norms about family ties can shape whether friends feel entitled to grieve and how openly they express it, even when the friendship was deeply significant. Similar experiences were described by other participants. Vilde reflected on how friendship losses often receive less recognition than family losses and worried that friends can become invisible in mourning processes. As she put it, “I think you are forgotten as a friend.” Her account illustrates how bereaved friends may struggle to have their grief socially acknowledged despite the significance of the relationship. Several participants had been important sources of support for the deceased, yet this role was not always recognised after the death. Nina, for instance, had been closely involved with her friend and the friend’s family, informally acting as next of kin because the family lived far away. After the death, contact with the family ceased, leaving her to grieve alone despite her earlier near role.

The interviews also show how isolation becomes central in unrecognised grief. Participants without contact with the deceased’s family described a lack of spaces where memories could be shared and the relationship acknowledged. Without others who had known the deceased, remembering became an individual and often silent task. Participants further described social distance in several directions. Some participants without a history of substance use described feeling disconnected from the deceased’s substance-using circles. Conversely, some participants with current or previous substance-use experience described feeling distant from family members or non-using friends. This mutual sense of being positioned outside parts of the deceased’s wider network contributed to a feeling of being on the margins in several relational spheres simultaneously. At the same time, when recognition from the family occurred, it carried deep significance. Bereaved friends who experienced openness, inclusion, or communication from the parents described this as profoundly validating. As Vegard said:

I feel that his parents have really been there for everyone in his life. That was the only thing that could have been missing, right—that sense of resolution that comes from the parents talking to you and acknowledging your care for him and his care for you. But we have had that. His parents have supported us, kept us informed, and shared things about his life that we didn’t know, which has helped us understand why this happened.

Fredrik described a particularly isolating form of grief. Although he had been one of the deceased’s closest friends and was among the last people to speak with him before the fatal overdose, he remained largely outside the family’s mourning process. Years later, he still described a lack of closure and a sense of carrying the story of his friend’s final days alone. Such recognition affirmed the relationship publicly as well as privately, and for some, strengthened bonds with the parents in a shared space of mutual support. Vegard noted that the parents acknowledged the role the friends had played, creating a sense of reciprocal responsibility: the friends wanted to support the parents just as the parents supported them.

For participants who lacked this form of recognition, grief became quieter, more private, and often more complicated. They described uncertainty about whom they could talk to, and whether their grief might seem inappropriate compared with the grief of parents or siblings. Others, especially those who had distanced themselves from the deceased before the death, felt a quiet sense of displacement, as their social position did not match the emotional weight of the loss. Without recognition, uncertainty and loneliness grew; with it, friends found space for shared mourning and mutual care.

### 3.3. Moral Ties and Relational Guilt

Guilt appeared in many of the interviews and was described as an integral part of the bereavement process. Participants talked about it in different ways. Some felt guilty for having reduced contact or withdrawing when the situation became difficult. Others felt guilty for staying closely involved, wondering whether their support had helped or might even have contributed to the challenges. Several also mentioned specific situations that remained important to them after the death, such as an unresolved disagreement, a decision made under pressure, or a moment when they wondered if they should have acted differently.

Ingrid described how she sometimes thought that if she had stayed closer in the relationship, the outcome might have been different. Although those around her reminded her that staying could have had negative consequences for her own well-being, she still struggled with the idea that her presence might have made a difference. Vilde, who had set boundaries to protect her own life, also spoke about feeling guilty after the death. She knew the decision had been necessary at the time, but found it difficult that she and her friend never had the chance to discuss it further. As she explained, “It was very tough to reject someone so close, and then he dies. Because that is what can happen.”

These experiences suggest that guilt was often tied to the relational expectations participants associated with friendship, such as loyalty, presence, and support. When participants were uncertain about how others might view their position or their role, feelings of guilt were more likely to remain private and less openly expressed. After the death, many revisited earlier decisions with heightened self-criticism, reconsidering boundaries that had seemed necessary at the time.

Guilt also influenced how participants thought about the past after the death. Several described revisiting earlier points in the friendship, weighing what they had done and what they might have done differently. The finality of death shaped these reflections: they could no longer talk with their friend, resolve misunderstandings, or reconsider past choices. While some acknowledged that, on a rational level, they knew the death was not something they could have prevented, they also expressed a lingering sense that they could or should have done more. Nina similarly questioned whether she had done enough for her friend. Despite years of involvement and support, she repeatedly returned to the question of whether she could have helped more. For her, grief became what she described as a “double sorrow”, consisting not only of the loss itself but also of persistent self-questioning and responsibility.

For some, guilt was linked to a hesitancy to speak openly about the friendship or the circumstances surrounding the death. This could be because they were unsure how others would respond, or because the relationship had involved difficult situations they preferred not to discuss. In such cases, guilt contributed to a quieter and more inward-facing form of grief. When participants experienced recognition or support from the deceased’s family or from others who had known the friend, their feelings of guilt were often met with understanding. In those contexts, decisions made during demanding periods were more easily seen as reasonable responses to a complex situation.

Overall, guilt appeared as one aspect of how participants experienced their grief. It was connected to the responsibilities they felt within the friendship and to uncertainties about past decisions. For many, it remained central to how they made sense of the loss and their role in the relationship.

### 3.4. An Existential Turning Point

Participants described the death of a close friend whose life trajectory and life experiences often resembled their own. The loss raised questions about life’s timing, personal vulnerability, and the kind of person one is or hopes to become. This went beyond sadness at the death of someone close and involved a more direct consideration of mortality and how familiar life paths can shift. A recurring pattern was identification: a sense that “it could have been me.” Vegard reflected that his friend “was not so different from me,” noting similarities in their family backgrounds and everyday lives. As he put it:

… you kind of lose someone who, in one way or another, could just as easily have been yourself. I don’t know, he wasn’t that different from me. He was one year younger than me, and we had quite a similar upbringing, as far as I understood. I have two brothers, he had one brother, parents who lived together, leisure activities, went through pretty similar schooling. That’s just how it is in Norway.

Existential reflection was also evident in other accounts. Charlotte described the loss of her closest friend as “losing a part of myself” and explained that the death influenced important life choices and how she wanted to live in the years that followed. For her, the loss became closely connected to questions of survival, responsibility, and future life direction. Similar reflections were expressed by Fredrik, who compared his own life with that of the deceased. Looking back, he questioned why he had been able to survive and build a different future while his friend had not. Reflecting on their shared experiences, he described an enduring sense that their lives could easily have unfolded differently. For him, the loss therefore became intertwined with questions of chance, vulnerability, and survival.

For several participants, these similarities made the loss feel uncomfortably close to their own lives. Such reflections appeared in different forms. For some, the loss led to a reassessment of priorities, and even a wish to make choices that signalled care for themselves and others. For others, the reflections stayed largely private. Mikkel, who had never used substances himself, said that public talk about “drug users” now felt personal; he worried that others might assume he had used as well, and that his grief reactions would surface. Some also felt constrained by the stigma surrounding substance use; speaking about the deceased risked exposing parts of their life they wished to protect or being inadvertently associated with substance use themselves. This made it harder to find safe places for their reflections and grief. Some participants described ways of expressing their reflections more openly. Vegard chose to talk publicly about his friend’s death and created both a performance and poetry based on their friendship. He explained that responses from others had generally been positive and that people seemed to understand the care and affection that shaped how he spoke about his friend:

When I talk about him, I am simply referring to a friend who was unlucky but did everything he could to put things right. I have not felt that anyone has reacted disrespectfully. I think it is also connected to the care and affection that is part of how I talk about him.

For Vegard, sharing these reflections was a way of presenting his friend as he knew him and to acknowledge the role the friendship had played in both of their lives.

Across accounts, these reflections were linked not only to age or life stage, but also to shared experiences, identities, and possible futures. For some, losing a close friend marked a clear turning point with visible changes in priorities or everyday choices. For others, it remained a quieter awareness that life is limited and that expected paths can shift. Across accounts, the emphasis was less on dramatic change and more on ongoing reflection on how to live in the wake of the loss.

## 4. Discussion

This study shed light on how drug-death bereavement is experienced in the context of friendship. Friendship is a chosen and continually negotiated relationship grounded in reciprocity and relative equality, rather than prescribed obligation or generational hierarchy (Dunbar, 2018). These characteristics formed an important part of how participants described and understood their bereavement experiences. Our findings suggest that relational features such as voluntariness, equality, and shared life stage were often intertwined with experiences of vulnerability, particularly when the pre-loss relationship involved strain and emotional labour. These findings resonate with previous studies showing that drug-related deaths often place substantial demands on close relationships (Reime et al., 2026; Titlestad et al., 2021a). Quantitative research likewise demonstrates that losing a close friend can have profound and enduring consequences, with studies reporting long-term reductions in both mental health and social functioning (Liu et al., 2019). Together, this evidence situates our findings within an emerging understanding that the quality and complexity of earlier relational dynamics, particularly in contexts involving substance-use difficulties, influence how traumatic loss may be experienced and interpreted.

Within this voluntary relational frame, participants described substance-use difficulties as introducing additional emotional, ethical, and relational labour. Friends often negotiate shifting degrees of closeness and distance (Roberts & Dunbar, 2011), movements well documented in research on family members affected by substance-use problems (Orford et al., 2010). Comparable research focusing specifically on friendships remains limited. As substance-use problems intensify, maintaining this relational balance becomes increasingly complex. Participants described different ways of navigating closeness and distance within the friendship. Some participants without substance-use experience described a growing need to create protective distance over time, whereas some participants with current or previous substance-use experience described continued everyday closeness and shared involvement in substance-use contexts (Selseng et al., 2023). Pre-loss decisions about closeness and boundaries appeared to influence how participants experienced the loss and reflected on it afterwards. Bereavement following the death of a friend carries a substantial psychosocial burden (Liu et al., 2019), and this burden may be amplified when the pre-loss relationship has been marked by ongoing and often demanding relational and ethical labour.

Friendship bereavement unfolds within a social landscape where grief is unevenly legitimised (Peskin, 2019) and disenfranchised (Doka, 2002). Culturally shaped grief hierarchies tend to prioritise family loss, leaving friends uncertain about whether their grief is socially warranted (Peskin, 2019; Robson & Walter, 2013). Our findings show that when families recognise the friendship and actively include friends in rituals or communication, it creates openings for shared meaning-making. When such recognition is absent, friends’ grief tends to become quieter, more solitary, and more difficult to articulate. These patterns align with quantitative studies indicating that the limited social support exacerbates the negative health effects of friend loss (Liu et al., 2019). Our findings extend this work by demonstrating how the need for social recognition interacts with the voluntary and often less publicly validated nature of friendships, shaping whether bereaved friends experience connection or isolation in the aftermath of a drug-related death.

Guilt is well recognised in bereavement research (Kennedy et al., 2019). In the present study, participants often linked feelings of guilt to friendship expectations concerning loyalty, presence, responsibility, and support. Given the voluntary and negotiated nature of friendship, decisions to create distance, remain closely involved, or continually recalibrate the relationship are often revisited with heightened self-scrutiny after the death. Previous studies of bereaved parents and siblings have highlighted concerns related to witnessing the suffering of other family members (Kalsås et al., 2023; Lindeman et al., 2023; Reime et al., 2026). In contrast, participants in the present study primarily focused on their own actions, limits, and obligations within the friendship prior to the death. Ethical and relational evaluations remained centred on the dyadic friendship itself, often involving retrospective reflections on responsibility, loyalty, and personal boundaries.

Participants frequently described existential reflections concerning mortality, vulnerability, survival, and future life direction following the death of a close friend. Participants’ reflections were not only linked to similarity in age. Across interviews, friends were often described as sharing biographies, identities, life trajectories, and imagined futures. The loss therefore disrupted not only a valued relationship but also an important component of participants’ understanding of themselves and their own future. In this sense, bereavement involved the loss of a shared life narrative as well as the loss of the friend. This was evident in participants’ reflections on survival, vulnerability, responsibility, and future life direction, illustrating how friendship losses can become intertwined with broader questions about who one is and how one wishes to live. Participants often described shared life stages, biographical parallels, and similarities in life trajectories as important aspects of losses they experienced as personally disruptive and closely connected to questions of identity, vulnerability, and future life direction. Participants’ reflections resonate with meaning-making models of traumatic and sudden loss, particularly regarding fairness, contingency, and vulnerability (Gillies & Neimeyer, 2006). This existential work unfolds alongside social positioning: some friends respond to stigma surrounding substance use by withdrawing into silence, reflecting the difficulties of making sense of a sudden and complex loss, while others seek to counter negative narratives through more active forms of storytelling or advocacy, efforts that can function as attempts at meaning reconstruction (Neimeyer, 2011).

Although several challenges described here, such as stigma, suddenness, and the complexity of pre-loss relational strain, align with broader literature on drug-related bereavement (Dyregrov & Selseng, 2022; Titlestad et al., 2021b), our study demonstrates how these processes unfold within the context of friendships. Quantitative studies show that even outside the context of drug-related deaths, the loss of a close friend can lead to prolonged psychological and social impairment (Liu et al., 2019). The present study extends this work by showing how the relational and ethical labour involved in supporting someone with substance-use difficulties, the marginal social position of friends within grief hierarchies, and the existential resonance of losing a close friend with whom one shares important life experiences, life-stage challenges, and imagined futures, intersect in participants’ accounts of drug-related bereavement and contribute to intertwined relational, social, and existential experiences. By bringing these dimensions together, the study offers a more nuanced understanding of drug-related bereavement and underscores the importance of recognising friends as a distinct group within trauma-related loss research.

### 4.1. Implications for Practice and Research

These findings point to the value of more intentionally involving friends in bereavement support and ritual practices. Participants described grief within friendships as shaped by reciprocity, shared experiences, and the absence of formalised social roles. Participants’ accounts suggest that acknowledgement from families and services may be especially important for bereaved friends, particularly when friendships are not automatically recognised within established hierarchies of grief. Our findings suggest that early communication, inclusion in funerals, and recognition of the friendship may help counter marginalisation and support shared mourning. Services working with bereaved families may also consider recognising friends as contributors to mutual support, for example, by inviting them into conversations about remembrance and practical arrangements where appropriate. Such practices may be particularly important following drug-related deaths, where participants described experiences of stigma, uncertainty, and limited recognition of their grief.

Participants’ accounts and emerging bereavement research suggest that digital forms of support may offer opportunities for recognition, shared remembrance, and connection for bereaved friends who remain outside family-based support structures. Online communities, digital memorial spaces, and peer-support initiatives may provide accessible and relatively private spaces for grieving and social support, while also presenting challenges related to privacy, emotional exposure, the quality of support provided, and potential overreliance on online interactions (Soh et al., 2026). Further research is needed to better understand their potential benefits and limitations.

Future research should attend to friends as a distinct group of bereaved individuals rather than treating them as an undifferentiated part of “family and friends”. Although research on friend bereavement remains limited (Liu et al., 2019), much of the existing literature has focused on adolescents and young adults (Chen et al., 2025). More research is needed to better understand bereavement experiences within adult friendships. There is also a need for studies that focus specifically on friends bereaved by drug-related death, given the additional challenges associated with stigma and the complex relational histories that often precede the death.

Studies that investigate how key features of adult friendship, such as voluntariness, reciprocity, and life-stage similarity, shape grief responses would offer important insights. Gender and life stage may influence how friendship grief unfolds, although these issues were not examined in the present study and warrant further investigation. Finally, future work should explore how assumptions about substance use and associated stigma affect whether friends feel able to speak openly about their grief and seek support.

### 4.2. Strengths and Limitations

This study reflects a specific social and cultural context, and the sample size limits the transferability of the findings. The sample was heterogeneous with regard to age, friendship histories, substance-use experiences, and time since loss. Participants reflected on bereavements that had occurred between one and 36 years previously, and the friendships represented diverse relational forms and social contexts. Such variation may have influenced both bereavement experiences and retrospective meaning-making. Participants’ relationships to substance use varied, but this dimension was not the focus of the analysis and could be examined in more detail in future studies. Although some patterns appeared to differ across participants with and without substance-use experience, the study was not designed as a comparative analysis and these observations should therefore be interpreted with caution. As with all retrospective accounts, participants’ reflections are shaped by the time that had passed since the death, and by the ongoing meaning-making that occurs as relationships evolve after loss. These factors should be taken into account when interpreting the findings. At the same time, the in-depth, situated accounts offered here provide insight into relational dynamics that have received little attention and highlight areas where more targeted and nuanced research is needed.

## 5. Conclusions

Bereavement following a drug-related death involves more than emotional distress: it is shaped by relational histories, social positioning, and existential questioning. Because friendships are voluntary, negotiated, and typically between peers, participants described grief within friendships as shaped by reciprocity, shared life experiences, and the absence of formalised social roles. Friends occupy a meaningful but often unrecognised position within bereavement contexts, and their grief is affected by cultural expectations around legitimate mourning, the emotional labour of maintaining complex relationships, moral evaluations of past decisions, and the existential impact of losing someone whose life has run closely alongside their own. Recognising these dimensions broadens understandings of bereavement and highlights the need to recognise friendship as a significant relational context, particularly in the aftermath of drug-related deaths.

## Figures and Tables

**Table 1:** Participants’ characteristics.

Participant	Gender	Age at Interview	Age at Loss	Substance-Use Background	Time Since Loss ^1^	Deceased’s Age at Death ^2^
Arne	Man	54	18	In treatment for substance-use problems	36	20–29
Ingrid	Woman	44	27	No history of substance use	17	20–29
Bengt	Man	49	33	Past substance-use experience	16	30–39
Charlotte	Woman	29	23	Past substance-use experience	6	20–29
Mari	Woman	32	21	Past substance-use experience	12	20–29
Jonas	Man	32	24	Current substance use	8	20–29
Vegard	Man	21	20	No history of substance use	1	20–29
Vilde	Woman	40	38	Past substance-use experience	2	30–39
Rune	Man	49	42	Past substance-use experience	7	30–39
Silje	Woman	43	37	Past substance-use experience	6	30–39
Mikkel	Man	24	23	No history of substance use	1	20–29
Olav	Man	28	27	Current substance use	1	20–29
Fredrik	Man	38	23	Past substance-use experience	15	20–29
Nina	Woman	39	36	No history of substance use	3	30–39

^1^ Time since loss at the time of interview; ^2^ Ages are grouped to protect anonymity.

## Data Availability

Restrictions apply to the datasets due to ethical and privacy considerations. The data presented in this study are available upon request from the corresponding author.
